# Virtue ethics and the commitment to learn: overcoming disparities faced by transgender individuals

**DOI:** 10.1186/s13010-019-0079-2

**Published:** 2019-08-22

**Authors:** Jennifer Markusic Wimberly

**Affiliations:** Parkland Health and Hospital System, Dallas, TX USA

**Keywords:** Transgender, Virtue ethics, Health disparities, Philosophy of medicine, Edmund Pellegrino

## Abstract

The purpose of this paper is to utilize virtue ethics as the appropriate paradigm by which to improve health care delivery to transgender individuals. Health disparities for transgender individuals occur external to the medical environment as well as internal to the medical profession. A commitment to virtue ethics should be undertaken to improve the care to transgender individuals.

In this manuscript I call on virtue ethics to address the intersectionality of such environmental structures for the promotion of the good of the patient as per the telos of medicine by Edmund Pellegrino, consistent with the eudaimonia of Aristotle’s Nicomachean Ethics. Virtue ethics is the appropriate paradigm for which bioethics can address the framework that poses barriers to access to health care and maintenance of health through a lack of competent, knowledgeable and compassionate providers for the transgender population.

Further, I pose that ascribing to improving the care to the individual transgender patient involves a call to action to overcome social ecological spheres of influence that are affecting the health of the individual and thereby the population of the transgender individuals as a whole. Through virtue ethics, the virtuous physician improves the health of the transgender individual and the character of themselves and the profession of medicine.

## Background

Health disparities of the transgender population exist external to the medical profession in the form of access to care as well as internal to the medical profession through a lack of knowledge by providers to provide comfort in compassionate and competent care. Further, these factors intersect in all aspects of the individual experience of the health care setting as the societal construct not only produces the environment providing such barriers to access through policies, legal protections, and insurance coverage, but also promotes the medical education that places conformity to a binary identity as normative.

## Main text

### Can teaching virtue overcome Transgender health disparities?

Health disparities of the transgender population have been outlined as external to the medical profession in the form of access to care as well as internal to the medical profession through a lack of knowledge by providers to provide comfort in compassionate and competent care. Further, these factors intersect in all aspects of the individual experience of the health care setting as the societal construct not only produces the environment providing such barriers to access through policies, legal protections, and insurance coverage, but also promotes the medical education that places conformity to a binary identity as normative, which I will term the epistemic barrier.

I here call on virtue ethics to address the intersectionality of such environmental structures for the promotion of the good of the patient as per the *telos* of medicine by Edmund Pellegrino, consistent with the *eudaimonia* of Aristotle’s *Nicomachean Ethics*. Virtue ethics is the appropriate paradigm for which bioethics can address the framework that poses barriers to access to health care and maintenance of health through a lack of competent, knowledgeable and compassionate providers for the transgender population.

**Fig. 1:**
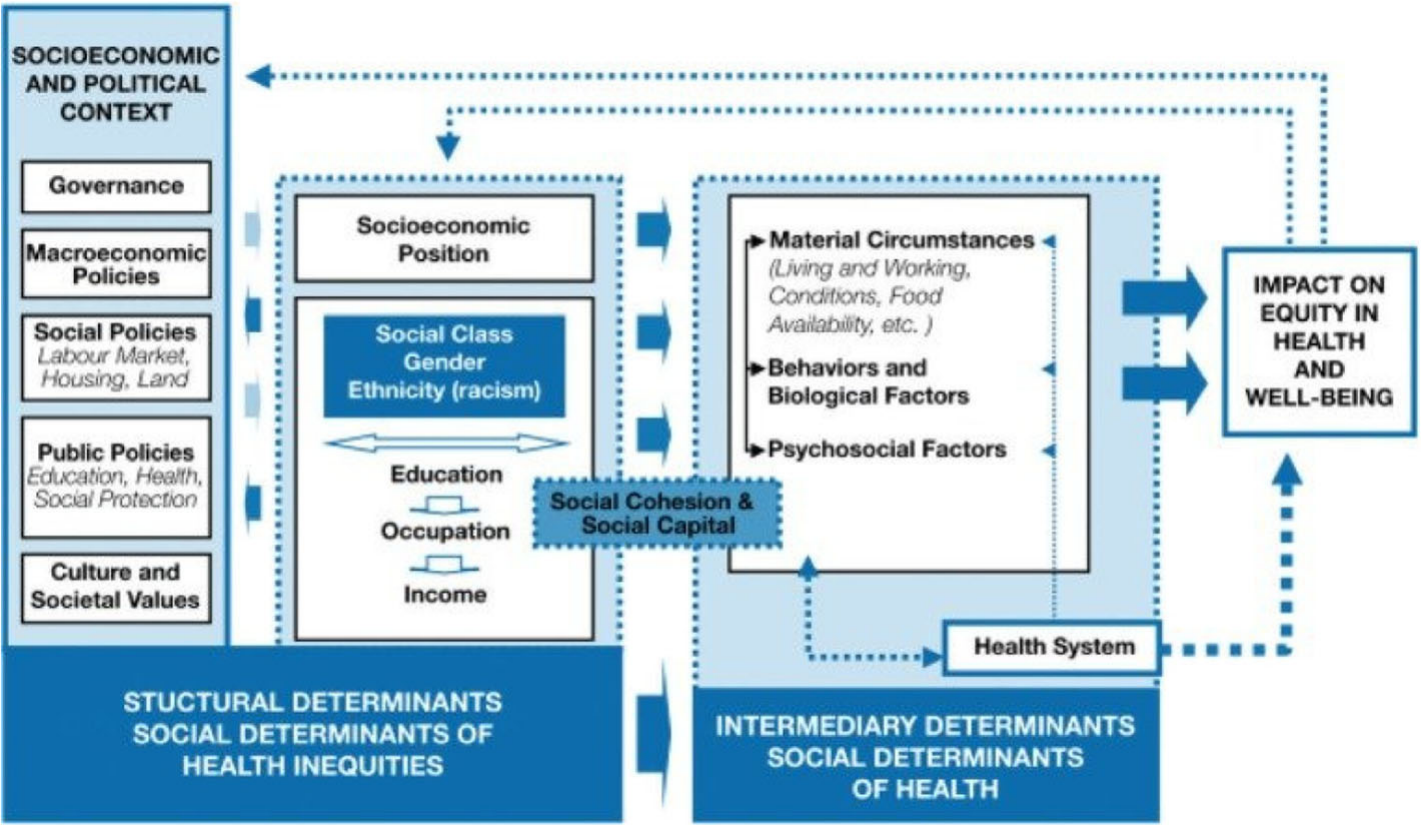
World Health Organization Commission for Social Determinants of Health Conceptual Framework, as depicted in the RAND Health Working Paper “Understanding the Upstream Social Determinants of Health”

Further, I pose that ascribing to improving the care to the individual transgender patient involves a call to action to overcome social ecological spheres of influence that are affecting the health of the individual and thereby the population of the transgender individuals as a whole. Medical sociology addresses this issue in the imagery of “upstream” focus as primary prevention with a narrative that while rescuing individuals drowning in a river it becomes necessary to focus upstream to see why they are falling in, as shown in Fig. 1 by the RAND Corporation [1] (Fig. 1).

This call to public policy engagement follows from Aristotle’s notion of *eudaimonia*, that happiness is the promotion of health for the society through health of the individual. The physician who engages in promotion of health of the individual will find themselves in the arena of overcoming barriers to the individual on the many levels of influence that affect the individual’s life and health and well-being. *Phronesis* or practical wisdom in medicine is the capacity to reason to attain the right end at the right time, to reach the mean, regarding the moral virtue of health. “Practical wisdom, then, must be a reasoned and true state of capacity to act with regard to human goods” [2]. Ronald Polansky holds that human good relates to the ultimate good, *eudaimonia*. In arguing for universality of *phronesis* in the setting of cultural diversity, Polansky finds human dignity is the universal natural virtue that binds moral activity for the virtuous person. “Virtue connects in a direct and convincing way with the notion of the dignity of the person, both the dignity of the virtuous person and the dignity of those with whom the virtuous person interacts. Virtue seems the true basis of human dignity” [3].

Once it is recognized that the commonality or universality of virtue can be found within the profession of medicine as the respect for human dignity, the question that must be addressed is if virtue can be taught. It would follow that in choosing the profession of medicine one is grounded in the ideals of the practice or art of such a life. Wayne Shelton holds that medical education grounds the profession in the ideals of the “good doctor” [4]. Edmund Pellegrino has previously outlined the “good of the patient” as the end of medicine and further stratifies this end into four concepts: the ultimate good, the biomedical or techno-medical good, the patient’s perception of his own good, and the good of the patient as a human person with the capacity to reason to make a choice [5]. To hold that virtue is a constant habitual disposition to reason to choose right action, Shelton offers practical approaches to virtue in medical education. This involves a call to developing the habits based upon modeling teachers in the profession, calling on Aristotle’s notion that the capacity to receive moral virtue involves natural virtue that must be reasoned through the habitual action of intellectual virtues. “Nature gives us the capacity of receiving them [virtues], and that capacity is perfected by habit” [2]. Virtue for Aristotle involves intellectual and moral excellence, with intellectual virtue originating in and being fostered mainly by teaching, whereas moral virtue is the outcome of habit [2].

Shelton holds that the profession of medicine calls for certain behaviors and dispositions and looks to medical educators to “embody and exemplify the habits of virtue in action” [4]. It is through respect for patients and professional behavior that virtue is honored by the medical profession and therefore virtue is exemplified, taught and becomes a habitual disposition, according to Shelton. An example of striking the mean and attaining the balance between the extremes of excess and deficiency that Shelton addresses is the student who fails to demonstrate a respect for the end of the “good doctor.” “What of the student who believes that such capacities are not essential to becoming a “good doctor”? Would we tolerate a student who was not interested in learning how to do a lumbar puncure” [4]? Here Shelton embraces the professionalism and the call to virtue that is integral to medicine as a good that must be honored on the equal grounding of technical skill, similar to the biomedical or technical good as defined by Pellegrino. The medical doctor is not only a technician, and though the profession calls for a necessary component of skill and technical ability and knowledge, the profession involves a disposition of character to promote the virtues of the profession itself. That is, the end of the profession of medicine is the good of the patient and the end of the professionalism in medicine is the good doctor. These are inalienable ends and virtue ethics is inseparable from medicine. Further, Pellegrino and Aristotle believe that virtues are able to be taught and in teaching virtue to physicians, Pellegrino maintains that to do so is “to safeguard the *telos* of the art” [6].

### Character and conscientious objection

To overcome the disparities in health care for the transgender individual through bioethics, focus on virtuous character formation of the physician will involve attention to education in the medical care of transgender people. For the purpose of teaching virtue there is a recognition of constant and universal natural virtue in the form of human dignity that provides support for such character development of the medical provider. This is for the end of medicine as well as of the medical profession and is exemplified by a constant habitual capacity to act in accord with right reason, to strike the mean between excess and deficiency. Maintenance of skill is necessary to promote the good of the patient, and skill, whether biomedical or technical, is one of the four concepts of the good of the patient according to Edmund Pellegrino.

Pellegrino, it must be noted, did not heed to all requests to respect the patient’s good. He was a fervent and prolific defender of conscientious objection. Physicians were free to withdraw from or refuse to provide care to the individual patient; “[i] n the event those goals are morally unpalatable to the physician, he is free to withdraw from the case under the usual conditions” [7]. Such conditions are to fulfill an obligation to not abandon the patient but to provide references, referrals or possible suggestions of other providers who may agree to participate in the patient attaining their goal [8].

It is this concept of conscientious objection that I must now turn. Shelton has addressed the intolerance of a medical professional who refuses to be educated in the art of what makes the “good doctor,” drawing resemblance to attainment of a technological or biomedical good. Taking Shelton and Pellegrino’s conditions together it can be acknowledged that the physician must agree to honor the dignity of the patient by agreeing to provide either care to promote the patient good, or, in times of a moral opposition to the goal as defined by the patient, to provide a resource for the patient to pursue the good by another provider. The profession of medicine is entrusted with an honor of refusal to abandon the patient.

If it is accepted that the doctor need not agree to treat the patient for whom there is a moral objection to the goal of the patient’s treatment, and it is accepted that the doctor carries a covenant to assist the patient in the pursuit of their goal to at least a minimum standard of obligation to refer to other providers or resources, the physician is in a position to maintain at least a degree of knowledge of possible resources for care to meet the patient’s goal. This is particularly salient in the multicultural and pluralistic society in which physicians practice medicine.

Reports such as the 2011 Institute of Medicine Report on the Health of Lesbian, Gay, Bisexual, and Transgender People as well as the US Transgender Survey provide an awareness of the health disparities of these individuals [9, 10]. Recognition of multiple layers of influence by which the disparities result includes a call to approach the policy and regulation to promote equity in the health for society’s individuals. The 2011 Institute of Medicine Report outlines barriers to health care access for the transgender individuals comprise multiple layers of life experiences of stigma from minority stress, intersectionality of multiple identities and social ecological perspective of spheres of influence [9]. Barriers to care also exist in a lack of provider knowledge and a social framework that embeds gender as a binary within medical education. Such inherent barriers within health care education propagate enacted stigma for the transgender individuals and further influence the above concepts of life experiences, intersectionality and social ecology perspectives. I challenge virtue ethics to address the individual while it must be recognized that the virtuous character of the physician also promotes the health of society, as per Aristotle’s *eudaimonia*. For the issue of conscientious objection, I will address the withdrawal from or refusal of care on both the individual and societal level as the issues are intertwined just as they are in accounting for health disparities.

Pellegrino recognizes the moral value of the relationship physicians and patients have on an individual basis as well as the covenant that binds together the two moral characters once a provider-patient relationship is formed. He calls upon the respect for the humanity of the patient and need to “allow him to make his own choices” [7]. But the moral agency of both participants in the provider-patient relationship does not bind the physician to participate in a good that conflicts with their own moral nature. Referring to differences in care regarding the healing relationship, Pellegrino writes “[w] hen conflicts occur in decisions involving human life – as for example with no-code orders, discontinuing life support measures, artificial insemination, abortion, etc. they often involve disagreements about the ultimate good and are therefore reconcilable with great difficulty if at all. Under such circumstances the patient-physician relationship should be respectfully and courteously discontinued since neither physician nor patient can morally compromise his belief system – particularly when the issue involves ultimate good” [7]. I will now examine the concept of gender in this light.

It is possible to envisage a transgender individual gaining access to health care after surmounting the barriers to care and encountering a provider who not only lacks the knowledge to provide compassionate care but also claims an inability to treat the individual based on the provider’s moral agency and belief. Basic health needs cannot refuse to be met by providers. “For example, one patient sought care for chronic kidney infections but was denied by multiple urologists once they realized the patient was trans. Providers typically cited ignorance about trans healthcare as the reason for denying care, regardless of whether or not patients’ concerns pertained to gender-affirming care” [11]. I will examine this narrative to illustrate how virtue ethics can approach the individual.

To this point I have acknowledged the character development of the physician through Aristotelian virtue ethics as comprised on utilizing reason to choose action that is right for the individual and for the profession of medicine. For the individual, the basic health needs of infection for a gender-affirming individual do not involve being “other than” an individual with an infection. The presence of identifying as a trans individual does not alter the relationship of healer to one needing healed, or in other words, the obligation or covenant of the caring professional to the patient in need of care. Such an individual should be met with technical skill as any other patient and the character of the “good doctor” would not permit a refusal of the individual’s health needs as “other” than what the virtuous physician treats.

Thus far in the examination of this narrative, the physician through habitual disposition of “being-at-work” is to meet the individual in need of treatment [12]. Recognizing a right to refuse to be involved in a patient’s care, it follows that in order to refuse to provide care, the physician must respectfully withdraw from the relationship. This is an obligation not to abandon the patient, or the concept of nonabandonment. The physician need not compromise one’s moral agency to act in accord with a compromise or concession of the other’s values but must recognize the good as the patient defines it is a value to the patient that must be recognized in congruence with the *telos* of medicine.

Noting that the medical condition of the individual in our narrative is contained within the *telos* of medicine, the restoration of the health and the good of the patient, an objection based on the gender identity of the patient is not congruent with the promotion of the professional as healer nor of the profession as one of healing. Beyond this concept of nonabandonment, it is the character of the physician that will not allow for the vice or self-interest of refusal to learn. Knowledge of the transgender vocabulary, awareness of the multiple contexts within which disparities arise and propagate, including the microaggressions of stigma enacted/realized, perceived/felt by provider’s lack of knowledge must be part of the work in action that is the character of the good doctor.

This serves to support the concept of the commitment to learn, to maintain skill. The physician may refuse to compromise to provide care to the transgender individual on the basis of an objection to their conscience or moral agency, however the profession and its involvement in the character of the good doctor does not permit the refusal to learn. The physician must not refuse to learn the knowledge of the vocabulary and the historical and social context by which transgender individuals face barriers to health care.

Therefore, on an individual basis, as we continue to envisage the physician refusal of care for the transgender individual in front of them, based on a moral objection and inability to compromise moral beliefs, the physician must not refuse to learn the contextual barriers that face the individual seeking care. The 2011 Institute of Medicine report demonstrates the multiple layers of overlapping constructs that the transgender individual faces to obtain and maintain health care. Stigma through enacted and felt/perceived minority stress, intersectionality of multiple identities which shape life experiences within a larger social context bind the “good doctor,” of good character by virtue of the respect for human dignity of all individuals, to be aware of these concepts that the individual will face in the pursuit of another provider to attain their definition of the “good.” The good doctor, recognizing the vice of self-interest, will honor their moral values by learning what barriers the patient faces and will strike the mean by providing the knowledge of available resources for the individual to utilize in gaining access to health care and competent, compassionate maintenance of health.

The medical issue of kidney infection in the sample narrative is an example of such overlapping structural barriers to restroom usage that transgender individuals face. Overt discrimination in refusal of access to a restroom and verbal harassment is reported in nine and 12 % of transgender respondents in the US Transgender Survey, respectively [10]. Such perceived/felt stigma contributed to more than half (59%) of transgender individuals avoiding using a public restroom, which consequently left nearly one-third (32%) of transgender individuals to limit the amount they ate or drank in order to avoid using public restrooms [10].

There is a call to action, through right reason, that the individual health needs may not be able to be met by the provider but will be acknowledged as a good to be pursued through other available resources. The doctor shall not simply state “I will not treat you,” but rather “I have available resources that you may pursue to assist you in treatment.”

Such a referral to competent compassionate care is an acknowledgement of the patient’s ideal of the “good” for themselves and a recognition of a technological or biomedical good that the physician is unable to provide. The skill may be one not possessed by the physician or may be one that the physician may not provide out of inability to compromise their moral values. But the virtuous physician meets the patient where they identify and maintains the skill of knowledge to assist the patient in the pursuit of health by recognizing disparities that are based on multiple complexities that the individual must navigate through society to obtain the “good” as the patient defines it.

“In the end, nonabandonment is a derivative obligation. It is grounded in the nature of the physician-patient relationship. This relationship is based on a promise to act always in the interest of the patient, and it calls not only for technical competency but also for advocacy of the well-being of the patient as perceived by the patient” [8].

#### Conclusion

The care of the transgender individual is contextually complicated by societal and institutional barriers, such as an epistemic gender binary based on sex assigned at birth that sets up discriminatory practices for basic health and well-being, such as use of the public restroom, with compounding abusive practices through interpersonal stigmatizing experiences. Further economic, employment and housing hardships serve as significant barriers to access to medical care and resultant health disparities.

The care provided to the transgender individual by the medical professional is another relational aggression based on the embedded medical knowledge of the same epistemic gender binary based on sex assigned at birth. The appropriate paradigm to undertake the challenge to overcome the overlapping contexts of determinants of health is bioethics in the form of virtue ethics, through character of the physician with the philosophy of medicine as the aim to achieve the good of the patient.

I have in this paper intended to evoke a knowledge of terminology as well as of disparities in health care outcomes for the transgender individuals that serves as the particulars to which the virtuous physician would achieve right action by choosing knowingly to overcome such obstacles. Knowledge develops the skill set which provides the comfort in care to fulfill the covenant as depicted by Edmund Pellegrino for the attainment of the good of the patient as they define it according to their life values and goals. Such judgment of practical wisdom or phronesis is virtue in action that shall improve the health of transgender individuals. In such activity in action, the physician will attain the moral virtue that does not constrain them but rather completes them “as a roof completes a house” [12]. Through virtue ethics, the virtuous physician improves the health of the transgender individual and the character of themselves and the profession of medicine.

## Data Availability

Not applicable

## Abbreviations

RAND: A nonprofit research organization
US: United States
